# Low Temperature Inhibits the Defoliation Efficiency of Thidiazuron in Cotton by Regulating Plant Hormone Synthesis and the Signaling Pathway

**DOI:** 10.3390/ijms232214208

**Published:** 2022-11-17

**Authors:** Hongmei Shu, Shangwen Sun, Xiaojing Wang, Changqin Yang, Guowei Zhang, Yali Meng, Youhua Wang, Wei Hu, Ruixian Liu

**Affiliations:** 1 Institute of Industrial Crops, Jiangsu Academy of Agricultural Sciences, Nanjing 210014, China; 2 Key Laboratory of Cotton and Rapeseed, Ministry of Agriculture and Rural Affairs, Nanjing 210014, China; 3 College of Agriculture, Nanjing Agricultural University, Nanjing 210095, China

**Keywords:** TDZ, low temperature, cotton leaf abscission, abscission zone, transcriptome, plant hormones

## Abstract

Thidiazuron (TDZ) is the main defoliant used in production to promote leaf abscission for machine-picked cotton. Under low temperatures, the defoliation rate of cotton treated with TDZ decreases and the time of defoliation is delayed, but there is little information about this mechanism. In this study, RNA-seq and physiological analysis are performed to reveal the transcriptome profiling and change in endogenous phytohormones upon TDZ treatment in abscission zones (AZs) under different temperatures (daily mean temperatures: 25 °C and 15 °C). Genes differentially expressed in AZs between TDZ treatment and control under different temperatures were subjected to gene ontology (GO) and Kyoto Encyclopedia of Genes and Genomes (KEGG) analyses to compare the enriched GO terms and KEGG pathways between the two temperature conditions. The results show that, compared with the corresponding control group, TDZ induces many differentially expressed genes (DEGs) in AZs, and the results of the GO and KEGG analyses show that the plant hormone signaling transduction pathway is significantly regulated by TDZ. However, under low temperature, TDZ induced less DEGs, and the enriched GO terms and KEGG pathways were different with those under normal temperature condition. Many genes in the plant hormone signal transduction pathway could not be induced by TDZ under low temperature conditions. In particular, the upregulated ethylene-signaling genes and downregulated auxin-signaling genes in AZs treated with TDZ were significantly affected by low temperatures. Furthermore, the expression of ethylene and auxin synthesis genes and their content in AZs treated with TDZ were also regulated by low temperature conditions. The upregulated cell wall hydrolase genes induced by TDZ were inhibited by low temperatures. However, the inhibition of low temperature on genes in AZs treated with TDZ was relieved with the extension of the treatment time. Together, these results indicate that the responses of ethylene and auxin synthesis and the signaling pathway to TDZ are inhibited by low temperatures, which could not induce the expression of cell wall hydrolase genes, and then inhibit the separation of AZ cells and the abscission of cotton leaves. This result provides new insights into the mechanism of defoliation induced by TDZ under low temperature conditions.

## 1. Introduction

Cotton is an important economic crop harvested for its natural fibers, and mechanized cotton picking has become the only way to progress the cotton industry. The technology of cotton leaf removal is an important prerequisite for realizing the mechanical harvesting of cotton, and the chemical defoliant is the core product of the technology. At present, thidiazuron (TDZ) (a synthetic cytokinin-like molecule) is the main defoliant of cotton [1,2,3]. The application of TDZ at the proper time before harvesting can promote leaf abscission, which can decrease the trash content and increase the fiber quality of machine-picked cotton [4,5].

The abscission process of plant organs is always divided into four steps [6,7]. Among them, the abscission signaling of abscission zones (AZs) is activated, and an enzymatic hydrolysis of the middle lamella in AZs are two key phases regulated by TDZ [8]. Plant hormones, ethylene and auxin, are important abscission signals regulated by TDZ [8,9,10]. TDZ induces the gene expression of ethylene biosynthesis and signaling in AZ, which results in the high production rate of ethylene [8]. Auxin has also been shown to modulate abscission. During leaf senescence, the level of auxin decreases alongside the increased production of ethylene [11]. TDZ downregulates auxin biosynthesis genes and transforms the auxin gradient from leaf to AZs [8]. The crosstalk between auxin and ethylene is one of the most important regulatory pathways in the control of abscission [12,13]. Cytokinin (CTK) and abscisic acid (ABA) are also affected by TDZ. TDZ affects CTK biosynthesis and metabolic genes to reduce indolepropionic acid (IPA) accumulation for the reduction in ethylene sensitivity, and TDZ regulates the gene expression of ABA biosynthesis and the signal and induces ABA accumulation [8].

When abscission is initiated by plant hormones, AZ cells begin to enlarge. Cell wall hydrolases promotes the degradation of the middle lamella and the loosening of the primary cell walls of separation layers [5]. Cellulase, polygalacturonase (PG), and pectinesterase (PE) are the three major cell wall hydrolases [14,15]. Defoliation can induce cellulase gene expression and increase the activities of hydrolases to promote leaf abscission [9]. TDZ can affect the expression of genes related to cell wall degradation in cotton AZs [8].

The efficiency of TDZ-induced cotton leaf abscission is affected by environmental conditions [2,16,17]. In order to ensure boll opening and good fiber quality, in China, the spraying time of TDZ is postponed for as long as possible, which results in this activity being performed at low temperatures during the defoliation period [18,19]. It has been reported that temperatures lower than 18 °C affect the efficiency of TDZ-induced leaf abscission [18]. Moreover, a low defoliation rate cannot meet the requirements of cotton mechanized harvesting. In this study, we firstly validate that the abscission rate of cotton leaves treated with TDZ is reduced by low temperature. Additionally, RNA-seq is performed to reveal the transcriptome change in cotton AZs induced by TDZ under different temperatures to determine the reason for why low temperatures affect the abscission of cotton leaves treated with TDZ. We demonstrate that low temperatures inhibit TDZs to induce plant hormone biosynthesis and signaling to control cotton leaf abscission. Understanding the response mechanism of cotton to TDZ under low temperature conditions can provide further information for this defoliation process, and then we can obtain some novel strategies to improve the efficiency of TDZ-induced cotton leaf abscission under low temperature conditions.

## 2. Results

### 2.1. The Defoliation Rate of Cotton Leaves Treated with TDZ Is Reduced by Low Temperature Conditions

**Figure 1:**
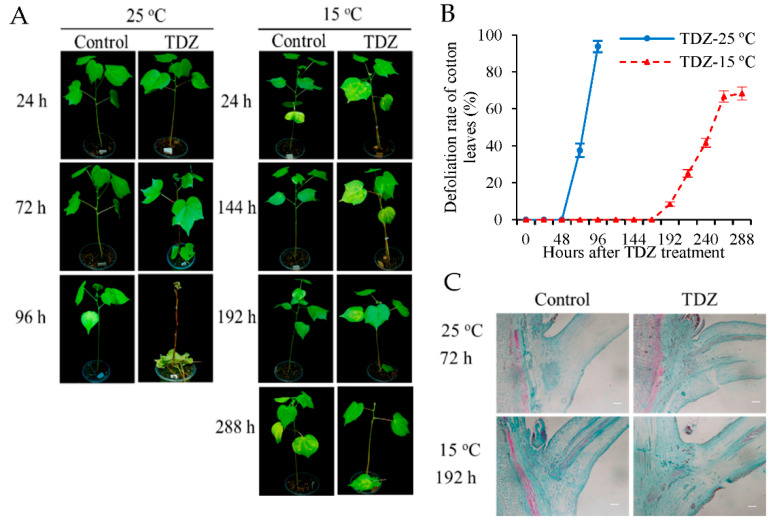
Effects of low temperatures on the abscission of cotton leaves treated with TDZ under different temperatures. (**A**) Representative images of cotton plants treated with thidiazuron (TDZ) or water (control) taken at 24, 72, and 96 h after treatment under 25 °C, and at 24, 144, 192, and 288 h after treatment under 15 °C; (**B**) defoliation rate (%) of cotton leaves at different time points after treatment. Error bars represent the SD of three biological replicates; (**C**) paraffin section observation of cotton AZs at 72 h after treatment under 25 °C and at 192 h after treatment under 15 °C. Scale bars are 200 µm. Cotton seedlings with the fifth true leaf fully expanded were used in the experiments.

To examine the influence of low temperature conditions on the defoliation rate of the cotton leaves, cotton plants treated with TDZ or water (control) were placed at 25 °C (normal temperature) and 15 °C (low temperature) conditions, respectively. Changes in the morphology were determined at 96 h (25 °C) and 288 h (15 °C) following TDZ treatment. The leaves remained green at 96 or 288 h following spraying with water under 25 °C and 15 °C conditions, respectively (Figure 1A). Leaf abscission was induced by TDZ. Under normal temperature conditions, the leaves treated with TDZ began to abscise after 48 h, and all leaves dropped at 96 h after treatment. However, under low temperature conditions, the leaves treated with TDZ began to abscise after 168 h, with a defoliation rate of ~65% at 288 h after treatment (Figure 1B). The peak of the defoliation rates at normal temperatures occurred at 48~96 h following TDZ treatment, while this occurred at 168~264 h after treatment under low temperature conditions. Additionally, the final defoliation rate following TDZ treatment in normal temperature conditions was significantly higher than that at a low temperature. This indicates that compared with normal temperature conditions, low temperature not only delays the time of cotton leaf abscission, but also decreases the defoliation rates induced by TDZ. Furthermore, the AZs of cotton plants treated with TDZ were examined by microscopy to elucidate the occurrence of cellular alterations (Figure 1C). Under normal temperature conditions, the cells of AZs treated with TDZ became separated at 72 h, while AZ cells of the control were well-organized. However, under low temperature conditions, the separation time of AZ cells treated with TDZ was delayed to 192 h, and it was consistent with the starting time of cotton leaf abscission. The results show that the separation of AZ cells induced by TDZ is affected by low temperatures.

### 2.2. Transcriptome Change in Cotton AZs Treated with TDZ under Low Temperatures

Based on the defoliation time of cotton leaves treated with TDZ under different temperature conditions, we selected AZs treated with TDZ and water (control) at 24 h under normal temperature conditions (N-24 h) and 24 and 144 h under low temperature conditions (L-24 and L-144 h) to perform the transcriptome analysis. The RNA-seq results show that more than 40 million clean reads for each sample were obtained (Table S1). The clean reads were mapped to the *G. hirsutum* reference genome. A total of 93–96% of reads were mapped in the tested samples, and 85–88% of the reads were uniquely mapping reads. The totally mapped reads were used to calculate the gene expression levels for the further analysis of differentially expressed genes (DEGs) [20].

**Figure 2:**
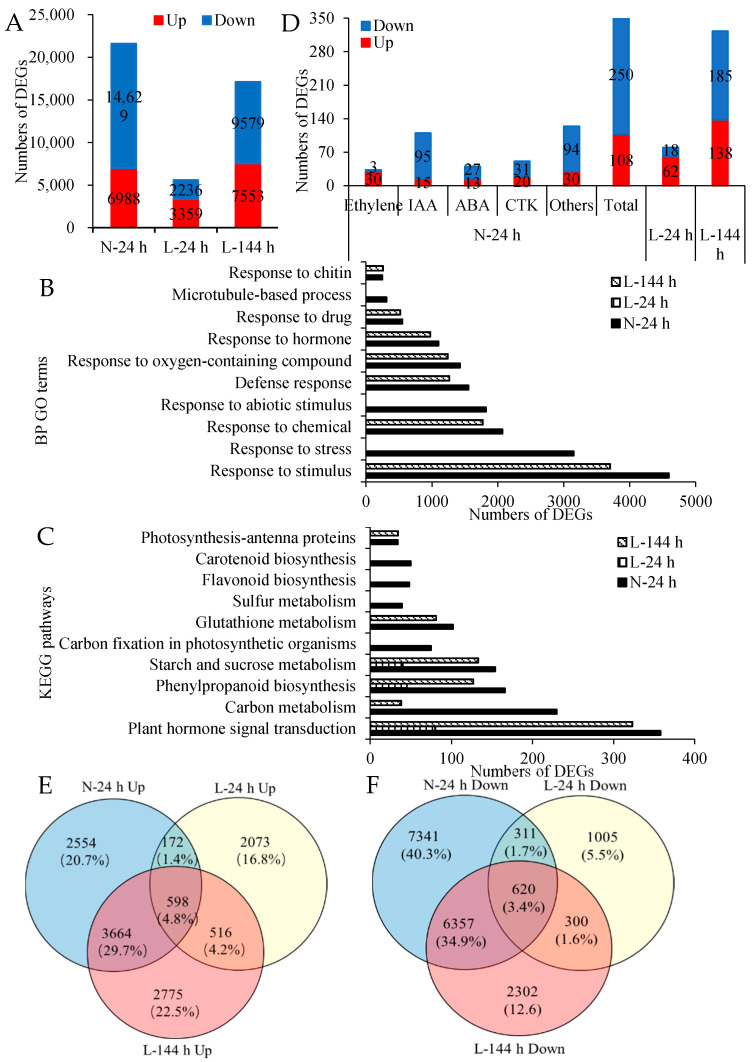
DEGs induced by TDZ (TDZ vs. control) in cotton AZs under different temperatures. (**A**) The numbers of DEGs in cotton AZs induced by TDZ. N-24 h, 24 h after treatment at normal temperature (25 °C). L-24 and L-144 h, 24 and 144 h, respectively, after treatment at low temperature (15 °C). The DEGs are controlled by *q*-value < 0.05 and |log_2_FC| ≥ 1. FC represents the expression-level fold change of genes. Black digits indicate the gene numbers; (**B**) enriched biological process GO terms (FDR (false discovery rate) < 0.001,). *X*-axis shows numbers of DEGs and *Y*-axis shows the top ten enriched GO terms of DEGs induced by TDZ at N-24 h; (**C**) enriched KEGG pathways (FDR < 0.05). *X*-axis shows numbers of DEGs and *Y*-axis shows the top-ten enriched KEGG pathways of DEGs induced by TDZ at N-24 h; (**D**) the numbers of plant signaling genes in cotton AZs regulated by TDZ; (**E**,**F**) Venn diagram displaying the number of DEGs unique to each time point or overlapping between different time points in AZs treated with TDZ compared with control. Upregulated DEGs (**E**). Downregulated DEGs (**F**). Black digits indicate the gene numbers. The percentage shows the proportion of DEGs of each part in all DEGs at three time points.

More than 60,000 genes were observed in the cotton AZs. Compared with the corresponding control group at two temperature conditions, 21,617 DEGs (6988 upregulated and 14,629 downregulated genes) were detected in AZs treated with TDZ at N-24 h, and 5595 (3350 upregulated and 2236 downregulated genes) and 17,132 DEGs (7553 upregulated and 9579 downregulated genes) were detected in AZs treated with TDZ at L-24 and L-144 h, respectively (Figure 2A). This result shows that TDZ induces less DEGs in AZs under low temperatures, but the number of DEGs increase with the extension of treatment.

To understand the functions of the DEGs, gene ontology (GO) terms enriched in the lists of DEGs were defined. They fall into three broad categories, comprising biological process (BP), cellular component (CC), and molecular function (MF). The top 10 BP enrichments (Figure 2B) showed that the DEGs induced by TDZ at N-24 h were ascribed to “Response to stimulus”, “Response to stress”, “Response to chemical”, and so on. This indicated that genes in AZs were activated at 24 h following TDZ treatment under normal temperature conditions. However, most of the top 10 BP enrichments of DEGs induced by TDZ at L-24 h were related to “cell wall biogenesis” or “metabolic process”. Comparisons among different temperatures revealed that there were no common GO terms between N-24 and L-24 h, while more than half of the top 10 GO categories of DEGs induced by TDZ at N-24 h appeared in the enriched GO terms of DEGs induced by TDZ at L-144 h.

To understand the DEGs involved in a specific pathway, Kyoto Encyclopedia of Genes and Genomes (KEGG) pathway analysis was performed. The DEGs induced by TDZ at N-24 h were enriched in “the plant hormone signal transduction pathway”, “carbon metabolism”, “phenylpropanoid biosynthesis”, “starch and sucrose metabolism pathway”, and so on (Figure 2C). Out of the top 10 KEGG pathways, only three appeared in L-24 h, but six pathways appeared in L-144 h. The GO and KEGG results show that the pathways induced by TDZ change under low temperature conditions.

The “Plant hormone signal transduction pathway” was the most enriched pathway of DEGs induced by TDZ at N-24 h, and there were 358 DEGs in this pathway. Among the 358 DEGs (108 upregulated and 250 downregulated genes) (Table S2, Figure 2D), 33 DEGs were related to ethylene (30 upregulated and 3 downregulated genes), 110 DEGs were related to auxin (15 upregulated and 95 downregulated genes), 51 DEGs were related to CTK (20 upregulated and 31 downregulated genes), 40 DEGs were related to ABA (13 upregulated and 27 downregulated genes), and the rest of the DEGs were related to other hormones. More than 90% of ethylene-signaling genes presented an upregulated expression, while 86% of auxin-signaling genes presented a downregulated expression. The results indicate that TDZ mainly increases the expression of ethylene-signaling genes and decreases the expression of auxin-signaling genes. These plant hormone signaling genes induced by TDZ were easily regulated by temperature. Under low temperature conditions, there were 80 and 323 DEGs in this pathway at 24 and 144 h following TDZ treatment, respectively. These results further suggest that the plant hormone signal transduction pathway responding to TDZ are negatively regulated by low temperatures, and the inhibition diminishes with the TDZ treatment time.

To determine the genes that respond to temperature, which might inhibit the effectiveness of TDZ treatment, Venn diagrams were used to analyze the DEGs induced by TDZ at different temperature conditions (Figure 2E,F). A small quantity of DEGs induced by TDZ overlapped between N-24 and L-24 h. The number of common upregulated DEGs between N-24 and L-24 h was 770 (172 and 598), which was approximately 11% of upregulated DEGs induced by TDZ at N-24 h, while the number of common upregulated DEGs between N-24 and L-144 h was 4262 (598 and 3664), which was approximately 61% of upregulated DEGs at N-24 h. The number of common downregulated DEGs between N-24 and L-24 h was 931 (311 and 620), which was approximately 6% of downregulated DEGs at N-24 h, while the number of common downregulated DEGs between N-24 and L-144 h was 6977 (620 and 6357), which was approximately 48% of downregulated DEGs at N-24 h. The result shows that under low temperature conditions, compared to 24 h following treatment, AZs treated with TDZ at 144 h presented more similar genes to those under normal temperature conditions, which indicates that the response of some genes to TDZ under low temperatures is delayed.

“Plant hormone signal transduction” was the most enriched KEGG pathway of the overlapped, upregulated DEGs induced by TDZ at N-24, L-24, and L-144 h. However, the most enriched KEGG pathway of the overlapped, downregulated DEGs induced by TDZ at N-24 and L-144 h and the downregulated DEGs induced by TDZ especially expressed at N-24 h was “Plant hormone signal transduction” (Table S3). The results further indicate that genes in the plant hormone signal transduction pathway present different responses to TDZ at low temperatures.

### 2.3. The Response of Plant Hormone Signaling Genes in Cotton AZs to TDZ Are Regulated by Low Temperatures

**Figure 3:**
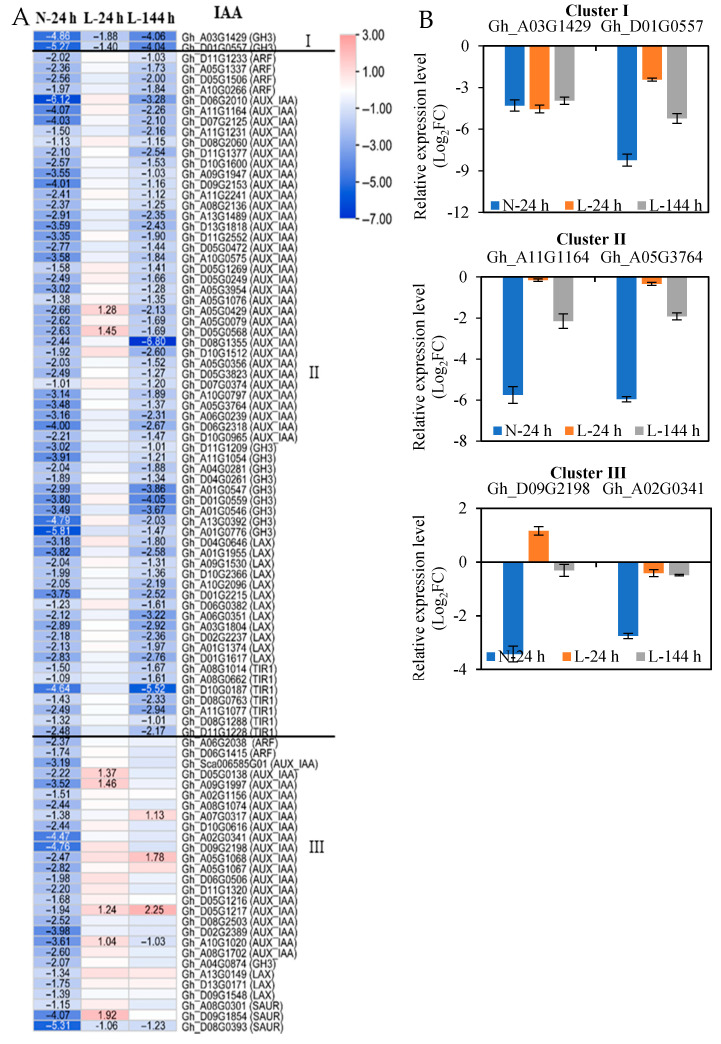
The expression of IAA-signaling genes in AZs treated with TDZ at different temperatures. (**A**) The heatmap is generated based on the RNA-seq data. The number in the box indicates the fold-change expression of the gene (only those with a ≥one-fold difference are shown). TDZ-induced downregulated and upregulated expressions are indicated by blue and red colors, respectively. I, II, and III represent three types, respectively. All the numbers in the heatmap represent fold changes; (**B**) the expression levels of selected genes from real-time quantitative PCR (RT-qPCR). The *Y*-axis indicates log_2_FC values. Data represent the means ± SD of three biological replicates, and each biological replicate contains three technical replicates. The same as below.

Among all DEGs in the plant hormone signal transduction pathway induced by TDZ, the number of indole-3-acetic acid (IAA) genes was the highest, and TDZ mainly downregulated their expression. However, these downregulated IAA-signaling genes presented different responses to low temperatures. Therefore, according to the response speed of genes to low temperatures, 95 downregulated IAA-signaling genes induced by TDZ at N-24 h could be divided into three clusters (Figure 3, Table S4). Among them, two IAA-signaling genes (two *GH3* (Gretchen Hagen 3) genes) appeared at three time points indicating the two G*H3* genes rapidly responded to TDZ at low temperatures (cluster I); 65 IAA-signaling genes appeared at N-24 and L-144 h indicating they slowly responded to TDZ at low temperatures (cluster II), and they contained 4 *ARFs* (auxin-response factor), 33 *AUX/IAAs* (auxin/indole-3-acetic acid), 9 *GH3s*, 12 *LAXs* (LIKE-AUX), and 7 *TIR1s* (transport-inhibitor response 1); and 28 IAA-signaling genes only appeared at N-24 h (cluster III) and they contained 2 *ARFs*, 19 *AUX/IAAs*, 1 *GH3*, 3 *SAURs* (small-auxin upregulated RNAs), and 3 *LAXs* (Figure 3A). Two genes from each cluster were selected to present the expression patterns with real-time quantitative PCR (RT-qPCR) (Figure 3B), and the expression of the six IAA-signaling genes indicated by RT-qPCR agreed well with the RNA-seq data. The results indicate that the majority of the downregulated IAA-signaling genes induced by TDZ are affected by low temperatures, and genes obtained from the same family have different sensitivity to low temperatures.

**Figure 4:**
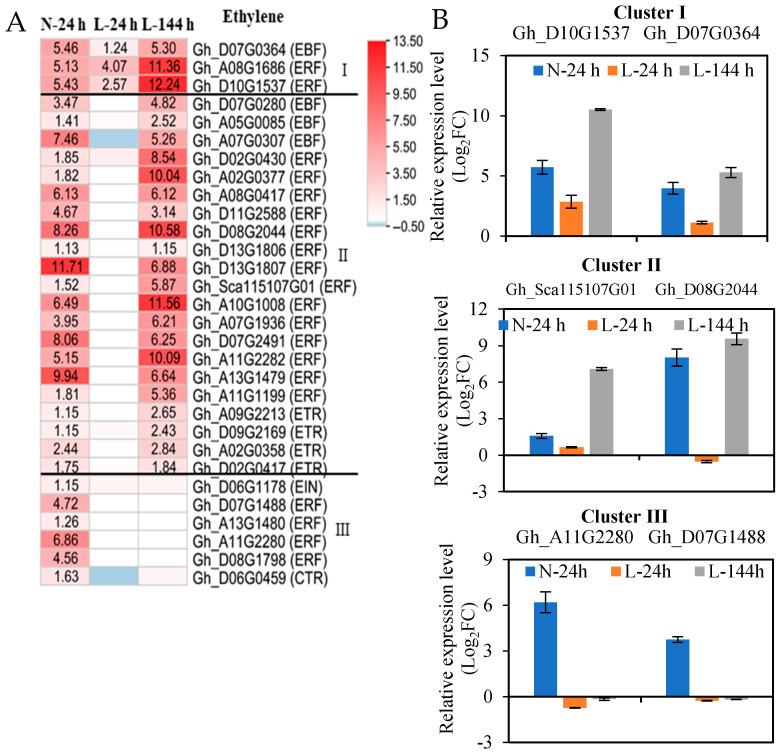
The expression of ethylene-signaling genes in AZs treated with TDZ at different temperatures. (**A**) Heatmap generated based on the RNA-seq data. The number in the box indicates the fold-change expression of the gene (only those with a ≥one-fold difference are shown). TDZ-induced downregulated and upregulated expressions are indicated by blue and red colors, respectively. I, II, and III represent three types, respectively. All the numbers in the heatmap represent fold changes; (**B**) the expression levels of selected genes from RT-qPCR.

TDZ mainly upregulated the expression of ethylene-signaling genes. A total of 30 upregulated ethylene-signaling genes induced by TDZ at N-24h were also divided into three clusters, according to the response speed of the genes to low temperatures (Figure 4, Table S5). A total of three ethylene-signaling genes (one EBF (EIN3-BINDING F-BOX) and two ERF (ethylene-response factors) genes) appeared at three time points (cluster I); 21 ethylene-signaling genes (3 EBF, 4 ETR (ethylene receptors), and 14 ERF genes) appeared at N-24 and L-144 h (cluster II); and six ethylene-signaling genes (one EIN3 (ethylene insensitive 3), one CTR1 (constitutive triple response 1), and four ERF genes) only appeared at N-24 h (cluster III) (Figure 4A). The expression patterns of two genes obtained from each cluster with RNA-seq and RT-qPCR (Figure 4B) were similar. The results indicate that low temperatures also affect the majority of TDZ-induced upregulated ethylene-signaling genes.

**Figure 5:**
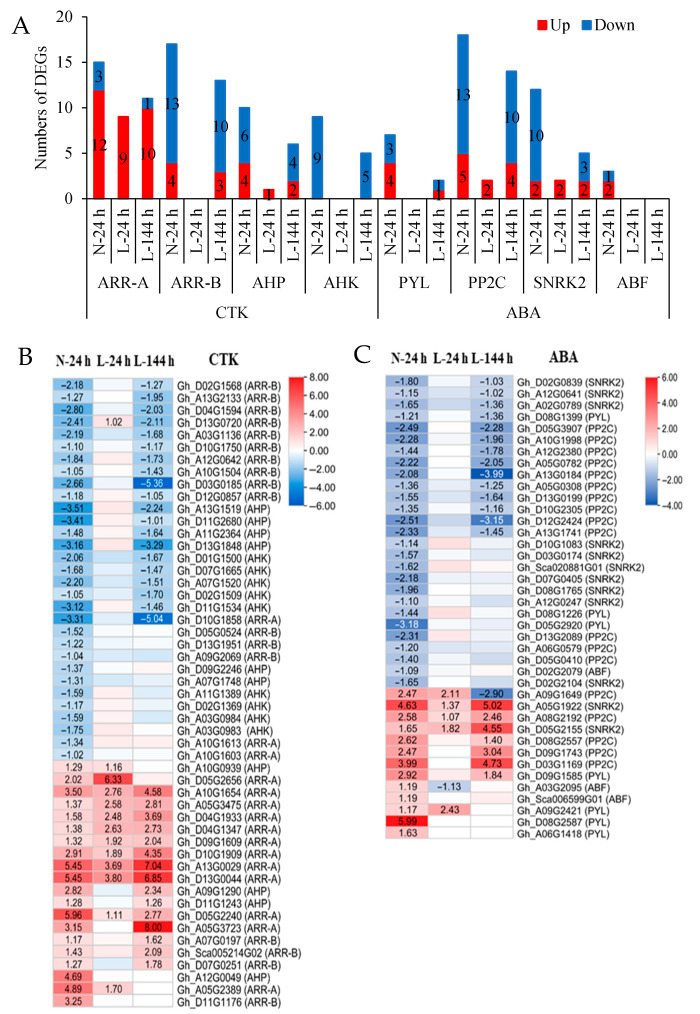
The expressions of CTK- and ABA-signaling genes induced by TDZ at different temperatures. (**A**) Numbers of DEGs; (**B**) heatmap of CTK-signaling genes generated based on the RNA-seq data; (**C**) heatmap of ABA-signaling genes generated based on the RNA-seq data. TDZ-induced downregulated and upregulated expressions are indicated by blue and red colors, respectively. The number in the box indicates the fold-change expression of the gene (only those with a ≥one-fold difference are shown). All the numbers in the heatmap represent fold changes.

The expressions of CTK-signaling genes (*AHK*, *AHP*, *ARR-B*, and *ARR-A*) were regulated by TDZ (Figure 5A,B, Table S6). Apart from *ARR-A*, the other three genes were mainly downregulated in AZs treated with TDZ. These downregulated genes were affected by low temperatures, and they were not induced by TDZ at L-24 h. At L-144 h, approximately 65% of downregulated CTK genes were induced by TDZ.

ABA-signaling genes (*PYL*, *PP2C*, *SNRK2*, and *ABF*) were induced by TDZ (Figure 5A,C, Table S7). *PYL* and *ABF* were mainly upregulated, while *SNRK2* and *PP2C* genes were mainly downregulated in AZs treated with TDZ. Apart from the upregulated *SNRK2* and *PP2C* genes, the other genes were not induced by TDZ at L-24 h. At L-144 h, approximately 50% of DEGs were induced by TDZ, but the expressions of *ABF*, *PYL*, and downregulated *SNRK2* in AZs treated with TDZ were significantly inhibited by low temperatures.

### 2.4. The Effect of Low Temperature on the Synthesis and Metabolism of Endogenous Plant Hormones in Cotton AZs Treated with TDZ

**Figure 6:**
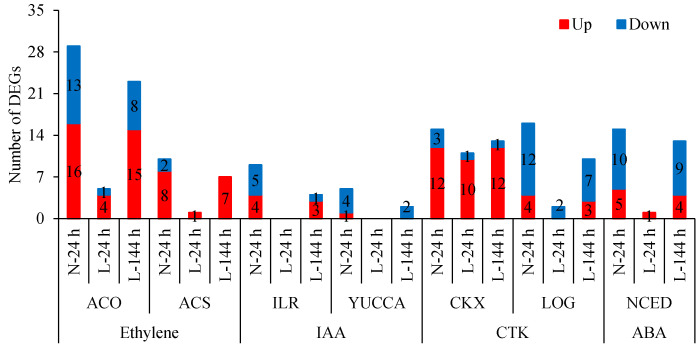
Numbers of plant hormone synthesis and metabolism genes in AZs treated with TDZ at different temperatures. The DEGs are controlled by *q*-value < 0.05 and |log_2_FC| > 1. Black digits indicate the gene numbers.

**Figure 7:**
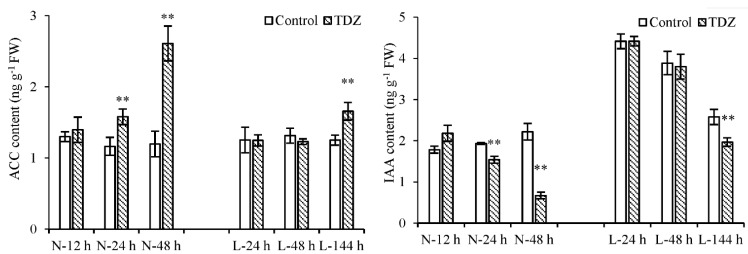
The contents of ACC and IAA in cotton AZs treated with TDZ and water (control) at different temperatures. Error bars represent the SD of three biological replicates. ** *p* < 0.01, Student’s *t*-test.

Apart from the plant hormone signal transduction pathway, the biosynthesis and metabolism of plant hormones play important roles in the abscission process. Ethylene-synthesis genes *1-aminocyclopropane-1-carboxylic acid oxidase* (*ACO*) and *1-aminocyclopropane-1-carboxylate synthase enzyme* (*ACS*) were differentially expressed upon TDZ treatment. Compared with the control, the expression levels of 29 *ACO* and 10 *ACS* genes changed following TDZ treatment at normal temperatures (Figure 6). Out of them, 5 *ACO* genes and 1 *ACS* gene were induced by TDZ at L-24 h, and 23 *ACO* and 7 *ACS* genes were induced by TDZ at L-144 h. Additionally, the numbers of upregulated *ACO* and *ACS* genes induced by TDZ were greater than those of downregulated genes. TDZ increased the 1-aminocyclopropane-1-carboxylic acid (ACC) content at 24 h under normal temperature conditions, while it increased at 144 h under low temperature conditions (Figure 7). This result indicates that ethylene-synthesis genes induced by TDZ are affected by low temperatures, and low temperature delays the time of ACC content increasing in TDZ-treated cotton.

IAA-amido hydrolase (*ILR*) and IAA-synthesis (*YUCCA*) genes were differentially expressed upon TDZ treatment. Compared with the control, the expression levels of nine *ILR* and five *YUCCA* genes changed following TDZ treatment under normal temperature conditions (Figure 6). Out of these, four *ILR* and two *YUCCA* genes were induced by TDZ at 144 h under low temperature conditions. No DEG was observed at 24 h at low temperatures. Additionally, the numbers of down-regulated *ILR* and *YUCCA* genes induced by TDZ were higher. Compared to the control, IAA content in AZs treated with TDZ decreased at 24 h at normal temperatures and at 144 h at low temperatures, respectively (Figure 7). Compared to normal temperature conditions, the low temperature affected the synthesis of IAA and delayed the time of IAA content decreasing in cotton AZs treated with TDZ.

Other hormone-related genes were also regulated by TDZ. The cytokinin dehydrogenase gene *CKXs*, responsible for CTK metabolism, was mainly upregulated, and the cytokinin-activation enzyme gene *LOGs* was mainly downregulated in AZs treated with TDZ (Figure 6), indicating that TDZ treatment could result in a decrease in CTK content in AZ. Compared to normal temperature conditions, 10 out of 12 upregulated *CKX* genes at N-24 h appeared at L-24 h, indicating that the response of *CKX* genes to TDZ was less affected by low temperatures. However, only 2 out of 16 *LOG* genes induced by TDZ at N-24 h appeared at L-24 h, indicating that the response of *LOG* genes to TDZ was significantly affected by the low temperature.

The ABA-synthesis gene (*NCED*) was mainly downregulated in AZs treated by TDZ, and only 1 gene appeared at L-24 h and 13 genes appeared at L-144 h (Figure 6). This indicated that the *NCED* gene was affected by the low temperature, but this effect decreased with the extension of the treatment time.

### 2.5. The Effect of Low Temperature on the Expression of Cell Wall Hydrolase Genes in Cotton AZs Treated with TDZ

**Figure 8:**
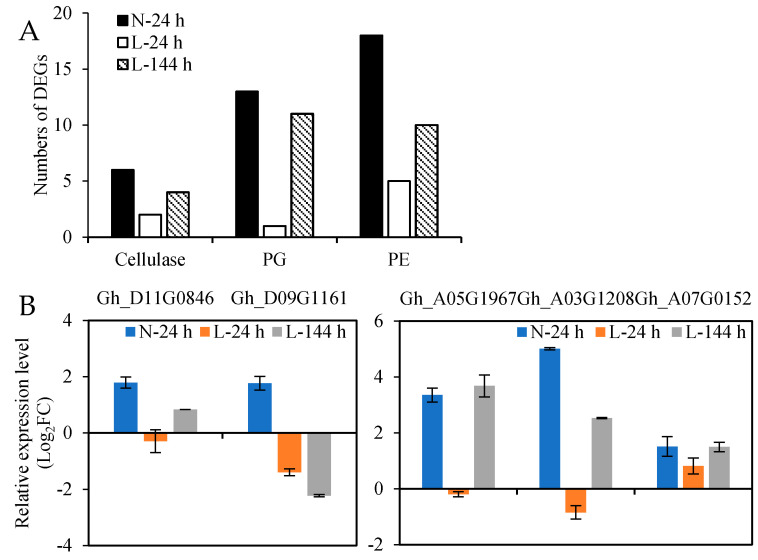
The expression of cell wall hydrolase genes in AZs treated with TDZ at different temperatures. (**A**) Numbers of upregulated cell wall hydrolase genes in AZs treated with TDZ; (**B**) the expression levels of selected cell wall hydrolase genes from RT-qPCR.

The third stage of the abscission process is the degradation of the cell wall and intercellular matrix; therefore, the upregulated cell wall hydrolase genes induced by TDZ might play important roles in this process. A total of 6 cellulase genes, 18 PE genes, and 13 PG genes were upregulated in AZs treated with TDZ at N-24 h, and most of them were affected by the low temperature. Among them (Figure 8A), eight genes (two cellulase, one PG, and five PE) were upregulated at L-24 h, and 25 genes (4 cellulase, 11 PG, and 10 PE) were upregulated at L-144 h. Five cell wall hydrolase genes were selected for RT-qPCR analysis (Figure 8B), and the results are consistent with the RNA-seq data. From the results, we can observe that 4 cellulase, 11 PG, and 6 PE genes slowly respond to TDZ at low temperatures, which might affect the time of AZs’ cell separation; one PG and seven PE genes were only upregulated in AZs treated with TDZ at normal temperatures, which might affect the defoliation rate of leaves under low temperature conditions (Table S8).

## 3. Discussion

TDZ, a widely used defoliant, has been attracting considerable attention in the field as it efficiently induces cotton leaf abscission [1]. However, its function is affected by environmental conditions, especially temperature. This study showed that low temperatures decrease the defoliation rates of cotton leaves treated with TDZ, and delay the time of leaf abscission. The decreased defoliation rates affected the fiber quality of mechanically harvested cotton [4,5].

AZ consists of small and dense cells interconnected by plasmodesmata, and then the AZ’s middle lamella was dissolved by hydrolytic enzymes, resulting in organ abscission [21,22]. The anatomical features of AZs were altered by leaf abscission accelerated by TDZ [1,3,5]. The result of this study shows that low temperatures delay the separation time of AZ cells treated with TDZ. The result of the transcriptome analysis shows that TDZ induces fewer DEGs in AZs, and the GO enrichments and KEGG pathways of these DEGs change under low temperature conditions. At normal temperatures, the GO enrichments of DEGs induced by TDZ are related to stress or chemical factors, but they are also related to cell wall biogenesis or metabolic processes at low temperatures. This result indicates that TDZ activates some genes at 24 h under normal temperature conditions, while the effect of TDZ is delayed and reduced at low temperatures.

Previous studies have shown that the biological process of TDZ-induced leaf abscission response is similar to abiotic stress-induced leaf abscission [23,24]. Several signaling pathways are involved in the control of organ abscission [1]. In this study, using RNA-seq analysis, the most enriched KEGG pathway of DEGs induced by TDZ was the plant hormone signal transduction pathway, indicating that TDZ promotes leaf abscission by regulating the plant hormone signal transduction pathway. Previous studies presented a similar result [8]. However, the response of genes in this pathway to TDZ was inhibited at low temperatures.

Cotton leaf abscission is initiated and regulated by ethylene [9], and the application of ethylene to these plants resulted in a rapid induction of organ abscission [25]. This study showed that the treatment of cotton seedlings with TDZ results in increased ACC content in AZ and upregulated expression of two ethylene-synthesis genes (*ACO* and *ACS*), indicating that TDZ induces ethylene biosynthesis and accumulation in AZs. Additionally, the expression of ethylene-signaling pathway genes (*EBF*, *ERF*, *ETR*, *EIN3*, and *CTR1*) in AZs was upregulated by TDZ, which could help ethylene induce its downstream gene expression to accelerate defoliation. At low temperatures, the expression of ethylene-synthesis and -signaling genes was affected. Among the genes induced by TDZ, only a few ethylene-synthesis (four *ACOs* and one *ACS*) and -signaling (one *EBF* and two *ERFs*) genes were induced at 24 h at low temperatures, while at 144 h, 15 *ACOs*, 7 *ACSs*, and 24 signaling genes (4 *EBFs*, 4 *ETRs*, and 16 *ERFs*) were induced. The results indicate that at low temperatures, most of the ethylene-synthesis and -signaling genes slowly responded to TDZ, which resulted in the fact that ethylene content present in AZs could not rapidly increase and it could not immediately induce the downstream genes. Previous studies [8] speculated that TDZ first induces *ETR* to perceive the ethylene signal and activate the positive regulatory-factor genes (*EIN2* and *EIN3*), promoting the expression of secondary transcription-factor ERFs, which ultimately promotes cotton leaf abscission. In *Arabidopsis*, ethylene-insensitive mutants *ETR1-1* and *EIN2* inhibit floral organ shedding [25]. Therefore, the inhibition of the response of ethylene-signaling genes (*ETR* and *ERF*) to TDZ at low temperatures results in the defoliation rates of cotton leaves being decreased and delayed. *EIN3* and *CTR1* were only significant differential expressions in AZs treated with TDZ at normal temperatures, which indicates that the two genes are key players in controlling ethylene transport at low temperatures.

The regulation of abscission also involves auxin, which functions as a brake, and a high auxin content in AZs inactivates organ abscission [26,27]. Our results show that auxin-synthesis (*ILR* and *YUCCA*) and -signaling pathway genes (*GH3*, *AUX/IAA*, *LAX*, and *SAUR*) are mainly downregulated in AZs treated with TDZ. The results indicate that TDZ reduces auxin biosynthesis, accumulation, and transduction in AZs. Auxin transporters regulate leaf shedding via the inhibition of polar-auxin transport in abscission zones [28,29]. At low temperatures, the response of IAA-synthesis and -signaling genes in AZs to TDZ was affected. Among the downregulated genes induced by TDZ, only two signaling genes were induced at 24 h at low temperatures, while at 144 h, 1 *ILR*, 2 *YUCCA*, and 65 signaling genes were induced. The results indicate that, under low temperature conditions, most IAA-synthesis and -signaling genes slowly respond to TDZ, which results in the IAA content not decreasing, and the auxin gradient is not formed. In this study, some IAA genes (4 *ILR*, 2 *YUCCA*, and 28 signaling genes) presented only a significant differential expression in AZs treated with TDZ at normal temperatures, which indicates that these genes might play important roles in controlling IAA content and transport at low temperatures. Previous reports determined that there was an interaction between auxin and ethylene in regulating organ abscission [12,13,30]. The application of IAA inhibited the expression of ethylene-induced genes in flower AZs [31]. In this study, the response of ethylene and IAA to TDZ was inhibited by low temperatures; therefore, both might be the key regulators for controlling the decreased defoliation rates of cotton leaves.

ABA and CTK were involved in the abscission process. Previous research shows that ABA advances abscission, but it does not directly affect AZ cells [32]. ABA affects flower AZs in *Lupinus luteus* by regulating the ethylene biosynthesis pathway [33]. The crosstalk between CTK and ethylene signaling plays an important role in cotton defoliation [1]. The suppression of *GhCKX3* inhibits ethylene synthesis and signal transduction, thus reducing the sensitivity to ethylene [34]. *CKX* was mainly upregulated in AZs treated with TDZ, so it might benefit ethylene synthesis; however, this gene was less affected by the low temperature. In this study, the response of some genes of ABA and CTK to TDZ was affected by low temperatures, but they did not necessarily act directly on AZ cells to regulate cotton leaf abscission.

The hydrolysis phase was controlled by cell wall-modifying enzymes [6,35,36]. Cellulase plays a major role in cellulose degradation. PE mainly hydrolyzes the methyl ester in the pectin and promotes the production of pectic acid. PG catalyzes the cleavage of 1,4-α-polygalacturonic acid in pectin molecules, which causes the cell wall to break [8,37,38]. In the process of cotton leaf abscission, the middle layer of AZs is hydrolyzed by cell wall-degrading enzymes, which are activated by an abscission signal [39]. In this study, the upregulated *cellulase*, *PG*, and *PE* genes induced by TDZ were affected by low temperatures. In AZ, *GhCel1* is ethylene-regulated and closely associated with the abscission of cotton leaves [9,40]. This indicated that low temperatures inhibited the increase in ethylene content, which could not induce the expression level of cellulase, PE, and PG genes, and then inhibited the hydrolysis of the cell wall and the abscission of cotton leaves. In this study, only one *PG* and seven *PE* genes presented a significant differential expression in AZs treated with TDZ at normal temperatures, which indicated that these genes might be key players in controlling the hydrolysis of the cell wall under low temperature conditions.

## 4. Materials and Methods

### 4.1. Experimental Design

A pot experiment was conducted in an artificial-climate laboratory. Cotton (CCRI50) seeds were selected and disinfected, and then seeded in pots (10 cm in diameter and 15 cm in height). When the cotton seedlings grew 5 true leaves, the following treatments were performed: temperature setting 25 °C (day/night 27 °C/23 °C, normal temperature, N) and 15 °C (day/night 17 °C/13 °C, low temperature, L), water, and TDZ application were set at each temperature treatment (the cotton was evenly coated with 0.1% TDZ using a brush). A total of 200 pots were planted for each treatment. The light source in an artificial-climate chamber was a biological sodium lamp; the light intensity was 450 ± 30 μmol·m^−2^·s^−1^, and the light time was 12 h.

Following treatment, the number of leaves were counted every day, and the defoliation effects were recorded by taking photos. Defoliation rate = number of shedding leaves/number of total leaves treated with TDZ. At 12, 24, 48, and 144 h following treatment, the abscission zone (AZ, 5 mm from petiole to distal end) of the third and fourth main leaves was sampled and stored at −80 °C for further use.

### 4.2. Histological Analysis

Histological observations were conducted to observe the structure of AZ cells. The samples were fixed in 70% FAA and dehydrated in alcohol xylene. They were then embedded in paraffin and cut into 8 μm thick sections and stained with 1% safranine and 0.5% solid green. Images were obtained using a light microscope (Nikon TE2000-S, Tokyo, Japan).

### 4.3. RNA Extraction and Sequencing

Total RNA was extracted from AZs using Trizol reagent (Invitrogen, Shanghai, China). RNA quality was assessed by Anoroad (Beijing, China) prior to library construction. mRNA was purified from the total RNA. Libraries were sequenced on an Illumina HiSeq platform (Anoroad, Beijing, China). The sequencing data generated in this study were deposited in the NCBI SRA database (BioProject: PRJNA892712). The cotton genome of *G. hirsutum TM-1* [41] was used as a reference genome, and the mapped sequenced reads were normalized to the aligned FPKM (expected number of fragments per kilobase of transcript sequence per million base pairs sequenced) to obtain the relative expression levels of the identified genes [20].

### 4.4. Analysis of DEGs

Differential expression analysis was performed using the DEGseq [42] R package. *p*-values were adjusted using the *q*-value [43], and a *q*-value < 0.05 and |log_2_(foldchange)| > 1 was set as the threshold for significantly differential expressions. Venn diagrams were generated using the function “Venn Diagram in R” based on the gene list of each sample. GO-enrichment analysis of the DEGs was performed using Blast2GO with FDR < 0.05. KOBAS 2.0 software with FDR < 0.05 was used to test the statistical enrichment of DEGs in KEGG pathways.

### 4.5. Plant Hormone Content

The endogenous hormones were extracted according to the previous research [44], and the contents of ACC and auxin (IAA) were determined by high-performance liquid chromatography tandem mass spectrometry.

### 4.6. RT-qPCR Analysis

Total RNA was extracted from AZs using an RNAprep pure kit (TSINGKE, Beijing, China). In preparation of conducting the RT-qPCR analyses, first-strand cDNA was synthesized using a SynScript™ III cDNA Synthesis Mix kit (TSINGKE, Beijing, China). The RT-qPCR reaction system and amplification process followed the manufacturer’s instructions of 2× TSINGKE^®^ Master qPCR Mix (SYBR Green I) (TSINGKE, Beijing, China). Analyses were performed on an ABI QuantStudio 5 Real-time PCR system (ABI, CA, USA). Three biological replicates were used for the RT-qPCR analyses. Relative expression levels were calculated following the 2^−ΔΔCT^ method using the *Actin* gene as an internal reference gene [45]. The primers used for RT-qPCR are listed in Table S9.

## 5. Conclusions

Our results clarify that the physiological and molecular mechanisms of low temperatures inhibit the rate of TDZ-induced cotton leaf defoliation. The plant hormone signal transduction pathway induced by TDZ was the key pathway affected by the low temperature. The response of ethylene and auxin biosynthesis and signaling pathways to TDZ were all inhibited by the low temperature, which could not induce the expression level of cell wall hydrolase genes, and then inhibited the separation of AZ cells and the abscission of cotton leaves (Figure S1). This study contributes novel information on improving our understanding about the application of TDZ to cotton defoliation at low temperatures, and identifies some candidate genes that might be key players in controlling the abscission of cotton leaves. However, how to efficiently increase the defoliation rate under low temperature conditions remains an important question in the research.

## Data Availability

Raw sequencing data can be accessed through the NCBI with the accession number PRJNA892712.

## Supplementary Materials

The following supporting information can be downloaded at: https://www.mdpi.com/article/10.3390/ijms232214208/s1.

## Abbreviations

ABA: abscisic acid; ACS, aminocyclopropane-1-carboxylic acid synthase; ACO, aminocyclopropane-1-carboxylic acid oxidase; AZ, abscission zone; CTK, cytokinin; DEGs, differentially expressed genes; PE, pectinesterase; PG, polygalacturonase; RT-qPCR, real time-quantitative PCR; TDZ, thidiazuron.
