# Supplementary figures and images for: Low Temperature Inhibits the Defoliation Efficiency of Thidiazuron in Cotton by Regulating Plant Hormone Synthesis and the Signaling Pathway
Source: Int J Mol Sci. 2022 Nov 17;23(22):14208. doi: 10.3390/ijms232214208 (PMC9694417; doi:10.3390/ijms232214208)

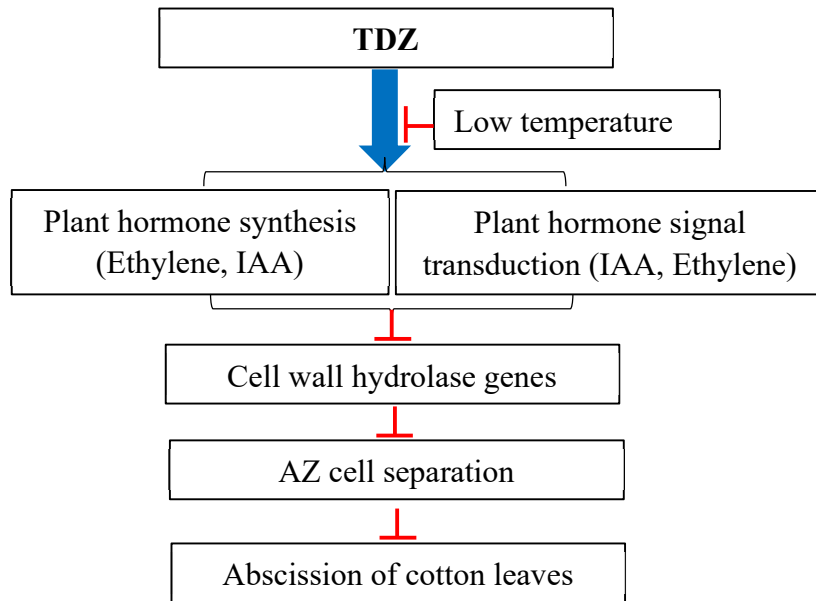

**Figure S1.** The pathway of low temperature inhibited the defoliation efficiency of TDZ in cotton.

Supplement: Supplementary file 1 [file ijms-23-14208-s001.zip › Figure S1.pdf]
